# Unraveling triacontanol impact in combating salt stress via enhancing growth, productivity, and physiological performance of wheat plant

**DOI:** 10.1186/s12870-026-08141-5

**Published:** 2026-02-09

**Authors:** Mervat S. Sadak

**Affiliations:** Botany Department, Agricultural and Biological Research Institute, National Research Centre, P. O. 12622, 33 El-Buhouth Street, Dokki, Giza, Egypt

**Keywords:** Antioxidant enzymes, Growth, Osmolytes, Salinity, Triacontanol, Wheat, Yield

## Abstract

**Background:**

Recently, the potential benefits of using triacontanol as a foliar treatment to mitigate the negative effects of salt on various crops have increased.

**Results:**

Salt-stressed wheat seedlings accumulated higher levels of phenols, hydrogen peroxide (H2O2), lipid peroxidation (MDA), osmolytes (proline, free amino acids and total soluble sugars TSS), and improved some antioxidant enzyme activities (catalase CAT, superoxide dismutase SOD, and peroxidase POD) compared with control plants. Meanwhile, salinity stress significantly decreased photosynthetic pigments, indole acetic acid (IAA) and nitrate reductase (NR) enzyme. All these alterations negatively affected growth characters and yield attributes in terms of shoot and spike length, shoot and spike weight/plant, grain weight/plant, and 1000 grains weight, as well as total carbohydrates of yielded grains. Meanwhile, triacontol treatments (25, 50, and 75 µM) lessened the reduced impact of salt stress on wheat growth and yield, moreover, treatment of triacontanol with 75 μM greatly enhanced the growth characteristics and yield attributes of wheat plants. Also, treatment with 75 μM triacontanol followed by 50 and 25 μM triacontanol resulted in improvements in photosynthetic pigments, IAA, greater phenols, TSS, proline, free amino acids, CAT, POX, SOD. While, H2O2 and MDA contents were decreased significantly in wheat plants treated with triacontanol, the highest decreases were obtained by 75 μM triacontanol foliar treatment.

**Conclusions:**

Our findings highlight the potential role of TRIA in mitigating the reduced impact of salinity stress and draw attention to the necessity of more study to fully comprehend the underlying mechanisms and investigate their usefulness in agricultural practices.

**Graphical abstract:**

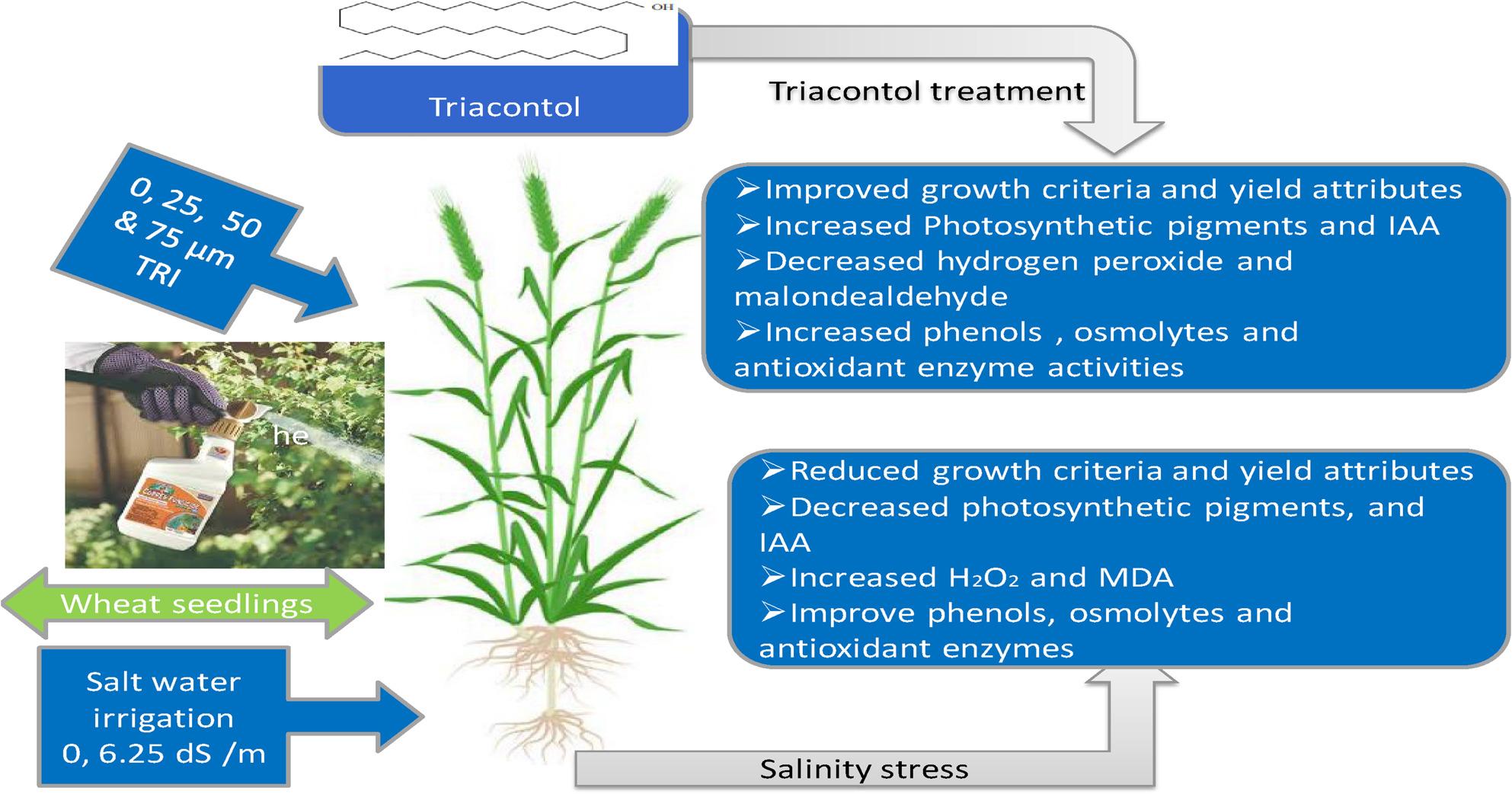

## Background

 Among the most valuable cereals all over the world is the wheat plant. Increasing wheat yield is necessary to meet the dietary needs of the world’s fastest-growing population [1]. Increasing wheat crop production through improving wheat varieties and agricultural practices is currently the main objective of the Egyptian government’s agriculture strategy [2, 3]. Plants being sessile are encountered with a variety of biotic (such as insects and viruses) and abiotic (such as cold, drought, flooding, heavy metal, and salinity) environmental disturbances. Among the various environmental stressors, salt is the most common environmental stressor that poses significant threats to agriculture as it affects plant growth, development, and yield [4]. Globally, salt affects over 6% of all agricultural soil [5], and this proportion increases daily because of a variety of anthropogenic and natural factors. Soil salinity can harm all stages of plant growth from seed germination to reproductive stage, leading to substantial crop yield losses [6]. Salinity caused osmotic stress, ionic toxicity, and secondary stresses, particularly oxidative stress [7], together with various morphological, physiological, biochemical, and molecular changes in plants, which directly inhibit plant growth and development.

Salinity causes overproduction of reactive oxygen species (ROS), which damages cells and their metabolic activities through oxidative stress. It also causes ion toxicity, disruption of plant metabolism, and membrane disarray [8]. Osmotic stress, which is really brought on by a water deficit, slows the growth of young leaves and new shoots in the short term by raising the concentration of salt (NaCl) to a threshold level. Water uptake, seed germination, cell elongation, leaf development, lateral branching, photosynthesis rate, nutrient uptake and translocation from root to shoot, abrupt supply of carbohydrates to meristematic tissues, and ultimately the plant’s overall growth are all impacted by osmotic stress [9]. Ion toxicity is then further produced by prolonged high salt concentrations in plant cells. Plants have a nutritional imbalance as a result of the disruption of the uptake of vital nutrients like Ca + and K + caused by ion toxicity of Na^+^ and Cl^−^. Furthermore, when an excess level of salt ions get into the plant’s transpiration stream damage plant cells by preventing photosynthesis and disrupting ion homeostasis [8], which further hinders the vegetative and reproductive growth of the plant. Because of the overproduction of reactive oxygen species (ROS) during metabolism, oxidative stress is brought on by the major stressors of osmotic stress and ion toxicity under salt stress. Overproduction of ROS can harm cells by causing lipid peroxidation, proteins, RNA and DNA molecules, and a breakdown in metabolic functions [10]. Overall, salinity is responsible for different types of stresses, mainly osmotic stress, ionic stress, and oxidative stress, which together obstruct physiological and biochemical activities of the plant, and result in impairing plant growth and development. In response to different types of environmental stressors, plants have evolved an effective and intricate process including morpho-physiological, biochemical, molecular, and anatomical variations to reduce salt stressors [11]. One of the strategies for improving stress tolerance is using plant growth regulators due to their important impact in plants.

Plant growth regulators are significant signal molecules either directly or indirectly regulate plant growth and development. Plant growth regulators also play an important role in the alleviation of abiotic stresses in plants via improving growth, development, nutrient allocation, and source-sink connections [12, 13]. The well-defined phytohormones that help plants cope with salt stress include auxins, gibberellins (GA_3_), cytokinins, nitric oxide (NO), ethylene (ET), brassinosteroids (BRs), and abscisic acid (ABA). Due to their significant role in plants, there is a need for seeking the new plant growth regulators and their effect in modulating the various metabolic processes in plants [14, 15]. Triacontanol (TRIA) is a relatively recent plant growth regulator, which modulates various physio-biochemical mechanisms causing alleviation of the negative effects of salinity in plants.

**Fig. 1:**
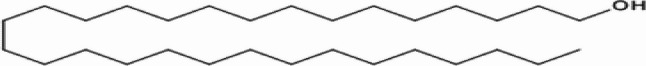
Chemical structure of 1-triacontol

Triacontanol is a naturally present saturated primary alcohol and potential plant growth regulator, early discovered in alfalfa hay and found in epicuticular waxes of plants [16]. Its chemical composition is C _30_H_62_O (Fig. 1).

**Fig. 2:**
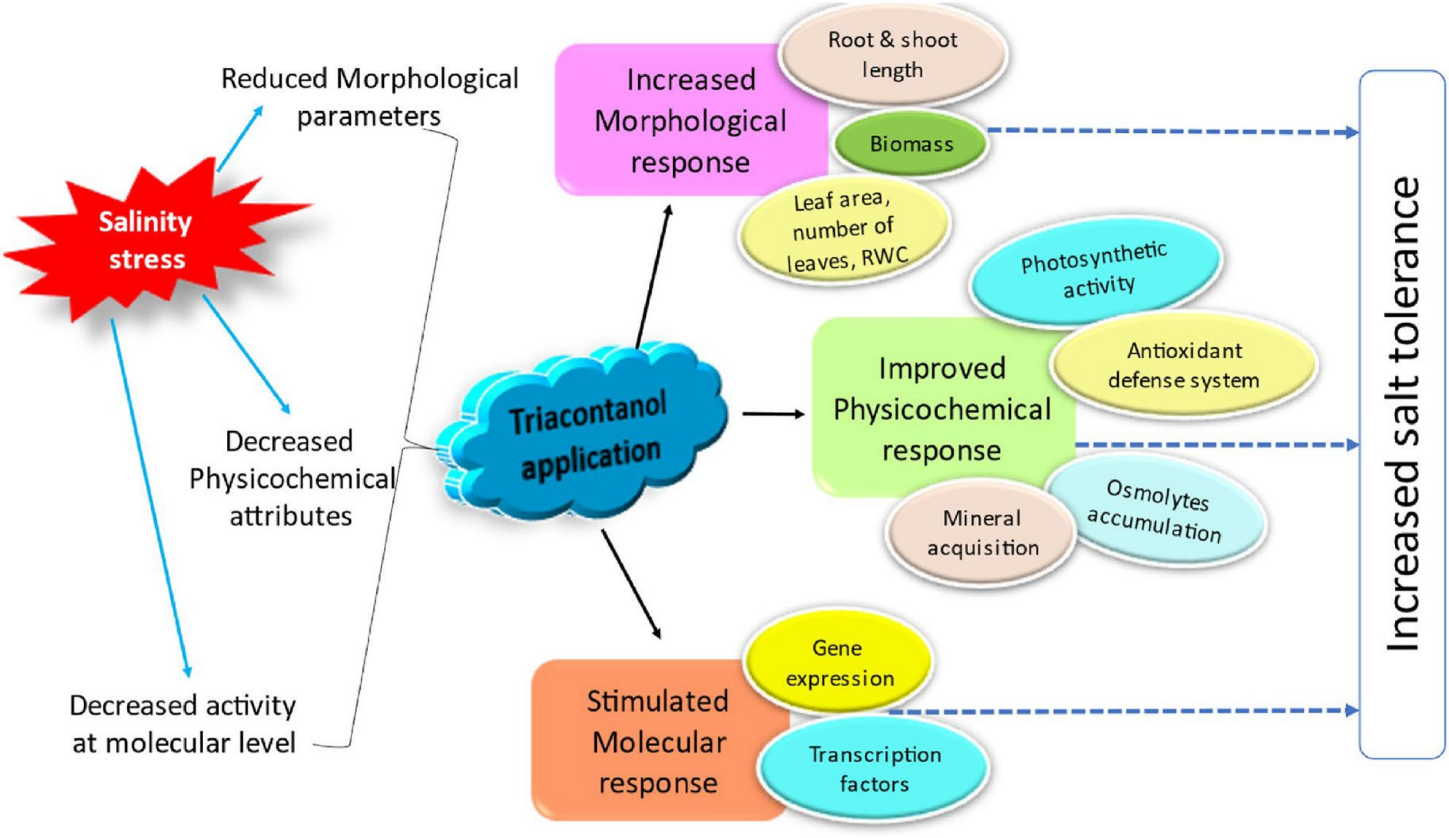
Diagrammatic representation of TRIA mediated salinity tolerance in plants (TRIA activates major metabolic pathways and increases the activity of different antioxidant enzymes responsible for detoxification of ROS, which in turn is responsible for the alleviation of salinity stress in plants) [24]

Its exogenous treatment promotes plant growth, nutrient and water uptake, photochemical pigments, photosynthetic rate, transpiration rate, internal carbon dioxide concentration, water use efficiency, carbonic anhydrase, nitrate reductase activity, sugars, soluble proteins, free amino acids, nitrogen fixation, crop yield, essential oil, and active constituents [17]. TRIA is mainly involved in regulating physio-biochemical processes of plants under varying environments, apart from its contribution to the up regulation of genes linked to defense. Triacontanol’s stimulating impacts have made it a fascinating compound for workers trying to boost plant output in different stressors, particularly salinity [18]. It has been employed in many plant species as an efficient growth regulator to promote shoot and root development and the production of secondary metabolites [19]. In saline environments, TRIA was showed to increase growth, photosynthetic pigments, & Ca^2+^ and K^+^ essential element concentrations intake [20]. Triacontanol gives plants the resilience to resist salinity stress and tolerate its negative effects [21]. However, studies showed that triacontanol was successful in reviving plant development under salinity stress conditions, along with increased photosystem II, chlorophyll content, CO_2_ fixation, and gas exchange characteristics [22]. By increasing metabolic activity during salt treatment, another study supported the beneficial effects of triacontanol on *Oryza sativa* photosynthetic efficiency with an increase in antioxidants under saline stress in soil. On the other hand, it has been discovered that applying TRIA exogenously to stressed plants—through foliar spraying, irrigation, soaking seeds, or adding it to the nutritional medium—elicits important mechanisms for tolerance to environmental stress [23]. Its exogenous application promotes the intake of water and nutrient as well as photosynthesis, nitrogen fixation, protein synthesis, amino acids, and antioxidant enzyme activity in plants (Fig. 2) [24].

Despite the advances, a significant knowledge gap exists regarding the use of triacontanol for salinity stress mitigation in wheat plants. The previous studies show that application of triacontanol has promising results on different plants against salinity stress condition. However, the mechanisms of underlying how triacontanol interact with plants have not been elucidated. Therefore, this study highlights the physiological role of triacontanol foliar treatment on wheat on alleviating the reduced impact of salt.

## Materials and methods

### Experimental procedure

Wheat (*Triticum aestivum* L. cv. Benisuef 5) grains were purchased from the Agricultural Research Centre, Ministry of Agriculture, Giza, Egypt. Triacontanol was obtained from Sigma–Aldrich Company. Salt was obtained from the Alex Trade Company, Egypt. The chemical constituents of salt content are moisture, 3.97%, purity (as NaCl), 98.02%, water insoluble matter, 0.05%, Ca^2+^, 0.21%, Mg^2+^, 0.11%, SO4^2−^, 1.40%, CaSO_4_, 0.71%, MgSO_4_, 0.54%, NaCl, 0.68%.

Some properties of the used soil prior to planting were determined [25] are pH 8,32, EC (dS/m): 1.35, the cations were Ca^2+^ 7,31, Mg^2+^ 3,00, Na^+^ 8,26 and K^+^ 1,80 meq L^1−^ while anions were HCO_3_^−^ 4,8, Cl^−^ 7,2, SO_4_^2−^ 8,36 meq L^1−^, particle size distribution (%), Coarse sand: 5.7, Fine sand: 17.3, Silt: 40.2, Clay: 36.8 and Texture: clay loam. The water holding capacity was maintained at about 90%. Following the guidelines provided by the Egyptian Ministry of Agriculture and Land Reclamation all guidelines’ practices related to wheat production were followed out. Wheat seedlings were thinned ten days after planting leaving four plants in each pot.

A pot experiment was conducted during the winter time of 2021/2022 and 2022/2023 in the National Research Centre’s greenhouse, Dokki, Cairo, Egypt (30̊ 20´ N, 31̊ 53´ E), the lowest and highest temperatures were 17.8 and 28.4 °C during the day and were 8.7 and 16.7 °C during nighttime. Daytime relative humidity ranges from 22.1 to 59.2% to explore the impact of Triacontanol (TRIA) on the growth and productivity of salt-stressed wheat. Wheat cv. Benisuif 5 grains with similar size and colour were selected and sterilized for nearly 2 min with 1% sodium hypochlorite and then rinsed in running water. Ten identical air-dried grains were planted in plastic pots containing seven kg of clay soil homogenized with sand with a 3:1 ratio (v/v). The physical and chemical properties of soil were determined. The soil had a clay-loam texture, with coarse sand at 1.4%, fine sand at 31.7%, silt at 39.6%, and clay at 27.3%, EC 1.82 dS m^−1^, pH 7.5, organic matter at 1.93%, CaCO_3_ 7.88%, and available N, P, and K accounting for 45.6, 7.8 and 415.0 mg kg^−1^, respectively. The soil was fertilized three days before planting with the following: ammonium sulphate (20.5% N) at an 800 kg ha^−1^ dose; super phosphate (15% P_2_O_5_) at a 240 kg ha^−1^ dose; and potassium sulphate (48% K_2_O) at a 120 kg ha^−1^ dose which was thoroughly incorporated into each pot. Following the guidelines provided by the Egyptian Ministry of Agriculture and Land Reclamation, all guidelines’ practices related to wheat production were followed. After emergence, the wheat seedlings were thinned ten days after sowing (DAS), and five plants per pot were left. Different TRIA concentrations were applied as foliar treatments twice after, 30 and 45 days from sowing, while salty water with two levels were added after second foliar application.

The experiment was factorial in a completely randomized design and replicated three times. The experiment comprised two factors. The first factor included two levels of sea salt at concentrations of 0.23 and 6.25 dS m^− 1^. The second factor involved Triacontol (TRIA) treatment with different concentrations (0.0, 25, 50, 75, and 100 µM). A preliminary germination experiment using different concentrations of TRIA (0.0, 5, 10, 15, 20, 25, 50, 75 and 100 µM) under different salinity levels (0.0, 1.25, 2.50, 3.75, 5.00, 6.25 ad 7.50 dS m ^− 1^) was conducted. Then, the appropriate concentrations of TRIA were chosen based on the results of growth characteristics. TRIA at the calculated concentrations were dissolved in distilled water and freshly used. To prepare the desire concentration of TR solution, hot distilled water with 0.1% tween 20 as a surfactant was used. TRIA different concentrations were foliarly treated two times at 30 ad 45 days from sowing. All spray treatments were completed early morning, before 9:00 a.m., with a hand sprayer at sufficient pressure to keep droplet size small. To obtain proper coverage, plants were sprayed from all sides. The spray volume amount of water consisted of approximately 500 L per ha based on each pot area being 0.049 m^2^. Plants were sprayed from both sides of the row to achieve adequate coverage. Pots were divided into two sets, every set watered by one of these salinity levels (0.23 dS m^− 1^ and 6.25 dS m^− 1^). Plants under salinity stress were watered two times by (liter/pot) with salinity water (6.25 dS m^− 1^) followed by one time with tap water. The plants were irrigated with equal volumes (one liter/pot) of tap water as well as the various salt solutions.

#### Measurements

Plant samples were obtained after 60 days of planting for record growth criteria data including shoot length (cm), leaves number/tiller, flag leaf area (cm2), tiller fresh weight (g/plant), root length (cm), root fresh weight (g), as well as some biochemical analysis. At full maturity (after 190 day from sowing) yield and its components: shoot length (cm), spike length (cm), shoot weight (g), spike weight/plant (g), grains weight/plant (g) and 1000 grains weight (g), in addition to carbohydrate percentage of the yielded grains.

### Biochemical analysis

#### Photosynthetic pigments

Using Li and Chen [26], method, total chlorophyll a and b and carotenoids (mg/g FW) contents in wheat (Benisweif 5 cv) fresh leaves were grounded and pestles by 80% acetone. Photosynthetic pigment levels were calculated as mg 100 g^-1^ fresh weight (FW) and calculated according to the following equations:

$$\text{Chlorophyll a}\;\left({\mu}\mathrm{g}\backslash\mathrm{mL}\right)=12.64\times\mathrm{A}665-2.99\times\mathrm{A}646$$

$$\text{Chlorophyll b}\left({\mu}\mathrm{g}\backslash\mathrm{mL}\right)=23.26\times\mathrm{A}646-5.6\times\mathrm{A}665$$   

$$\text{Total Chlorophyll}\left({\mu}\mathrm{g}\backslash\mathrm{mL}\right)=7.04\;\times\mathrm{A}665+20.27\times\mathrm{A}646$$ 

$$\mathrm{Carotenoids}\;\left({\mu}\mathrm{g}\backslash\mathrm{mL}\right)=\frac{1000\;\times\;\mathrm{A}470\;+\;0.89\;\times\;\text{Chl a}\;-\;52.02\;\times\;\text{Chl b}}{245}$$

#### Indole Acetic Acid Content (IAA)

IAA was determined by Gusmiaty [27], . A known weight of the fresh samples was taken and extracted with 85% cold methanol (v/v) for three times at 0 °C. The combined extracts were collected and made up to a known volume with cold methanol. Then take 1 ml of the methanolic extract and 4 ml of PDAB reagent (para-dimethylamino benzoic acid 1 g dissolve in 50 ml HCl, 50 ml of ethanol 95%) and left for 60 min in 30-400 C. The developing colour was spectophotometrically measured at wave length of 530 nm and expressed as µg/100 g fresh weight.

#### Phenols contents

Phenols were extracted ad estimated using Gonzalez [28]. A known weight of fresh sample (1 g) was extracted using 85% cold methanol (50 ml v/v) for three times at 90 °C. The combined extracts were collected and made-up to a known volume with cold methanol. Then one ml of the extract was mixed with 0.5 ml Folins - Ciocalteu reagent, shake, and allowed to stand for 3 min. Then 2 ml of saturated sodium carbonate (Na_2_CO_3_) was added to each tube followed by distilled water shaken and left for 60 min. The absorbance was determined at 750 nm using spectrophotometer and expressed as mg tannic acid 100 g^_1^ dry weight.

#### Hydrogen peroxide

The Hydrogen peroxide level was determined calorimetrically according to Velikova [29]. H_2_O_2_ was extracted from plant tissues by using acetone. Titanium reagent and ammonium were added to the extract and dissolved in sulphuric acid (1 M). The intensity of the yellow color of the supernatant was measured at 415 nm. The H_2_O_2_ concentration was expressed as nmol g^− 1^fresh weight.

#### Lipid peroxidation

Lipid peroxidation levels were expressed as malondialdehyde (MDA) level which was determined by method provided by Hodges [30]. A sample (200 mg) was homogenized in 10 ml of 0.1%trichloroacetic acid (TCA). 1 ml of the supernatant was centrifuged at 15,000×g for 20 min to aliquot of the supernatant 4.0 ml of 0.5% (w/v) thiobarbituric acid (TBA) in 20% TCA and 10 µl of butylated hydroxyl toluene (BHT) (4% in ethanol) were added. The mixture was heated at 95 °C for 30 min and then quickly cooled in an ice bath, centrifuged at 10,000×g for 10 min, and then the absorbance of the supernatant was recorded at 532 nm by spectrophotometer (VEB Carl Zeiss). The value for non-specific absorption at 600 nm was subtracted. The TBARS content was calculated using its absorption coefficient of 155 nmol^− 1^ cm^− 1^ and expressed as nmol (MDA) per g fresh weight.

### Assay of enzymes activities

Enzyme extractions were collected following the method described by Chen and Wang [31]. Leaf tissues were homogenized in ice-cold phosphate buffer (50 mM, pH 7.8), followed by centrifugation at 8,000 rpm and 4 °C for 15 min. Supernatant was used immediately to determine the activities of enzymes.

#### Assay of superoxide dismutase activity

Superoxide dismutase (SOD) (EC 1.12.1.1) activity was assayed at 560 nm by nitro-blue-tetrazolium (NBT) reduction method using spectrophotometer [31]. The reaction mixture (3 ml) was composed of 150 µ riboflavin (13 µM), 250 µ NBT (63 µM), 2.5 ml methionine (13 µM), 50 µ phosphate buffer (50 mM, pH 7.8), and 50 µ enzyme extract. One unit of SOD activity (1 nmol of substrate oxidized per minute) was described as the quantity of enzyme protein required for prevention of the 50% decrease of NBT.

#### Assay of peroxidase activity

Peroxidase (POX) (EC 1.11.1.7) activity was determined by Kumar and Khan [32]. 0.5 ml of the enzyme extract, 2 ml of 0.1 M phosphate buffer (pH 6.8), 1 ml of 0.01 M pyrogallol and 1 ml of 0.005M H_2_O_2_ were mixed. The solution was incubated for 5 min at 25 °C after which the reaction was terminated by adding 1 ml of H_2_SO_4_ (2.5 N). Purpurogallin amount formed was determined by measuring the absorbance at 420 nm using spectrophotometer [33].

#### Assay of catalase activity

Catalase (CAT) (EC 1.11.1.6) activity was determined spectrophotometrically by following the decrease in absorbance at 240 nm [33]. The mixture (3 ml) contained 1.9 ml phosphate buffer (50 mM, pH 7.0), 100 µl enzyme extract, and 1 ml0.3% H_2_O_2_. The reaction was initiated by adding enzyme extract. One unit of CAT activity was defined as the 0.01deduction in absorbance at 240 nm per minute. The enzyme activities were calculated by Kong [28]. Different enzyme activities were expressed as U min^− 1^ g^− 1^ fresh weigh.

####  Assay of Nitrate Reductase (NR) activity

The activity of nitrate reductase (NR, EC 1.7. 1. 1): Nitrate reductase (NR) was extracted as described by Foyer [34]. NR was measured according to Jaworski [35]. NR activity was expressed as nmole NO_2_ g^− 1^ fresh weight h^− 1^.

### Proline

Proline was determined by the method of Versluses [36], . After being pulverised to 0.5 g using 10 cc of 3% sulphosalicylic acid, the leaves were centrifuged at 10,000×g for 10 min. 2 ml of the supernatant and 2 ml of recently made acid ninhydrin reagent were combined. For thirty minutes, the mixture was incubated at 90 °C in a water bath. After stopping the reaction, it was chilled in an ice bath. To extract the reaction, 5 ml of toluene were added, and the mixture was vortexed for 15 s. The toluene and aqueous phases were then separated by 20 min in darkness. After gathering the toluene phase, the color’s absorbance was measured at 520 nm using proline as a standard, and the result was reported as mg 100 g^− 1^ dry weight.

### Free amino acids

The free amino acids were determined according to the method described by Sorrequieta [37]. Leaves were homogenized in ethanol (80%), then boiled for 10 min. and centrifuged at 2000×g for 10 min. 0.05 ml of the obtained supernatant and 2 ml of ninhydrin reagent were mixed and boiled in the water bath (15 min.). The mixture was kept at room temperature for cooling and was made up to 10 ml using distilled water. The color developed read at 570 nm using glycine as a standard using Spectro-colorimeter, the results were expressed as mg 100 g^− 1^ dry weight.

### Total soluble sugars

Total soluble sugars (TSS) were extracted according to Homme [38] and quantified using the procedure outlined by Chow and Landhausser [39]. After homogenizing a known weight (1 g) of dry tissue in 10 ml of 80% ethanol (v/v) at 25 °C and periodically shaking it overnight, the mixture was centrifuged at 6000×g. After being dried, the supernatant was dissolved in a predetermined amount of distilled water.0.1 ml of the extract was combined with 3.0 ml of freshly prepared anthrone (150 mg anthrone + 100 ml 72% H_2_SO_4_) for TSS analysis. The mixture was then boiled in a water bath for 10 min, cooled, and the absorbance was measured at 625 nm using a Spectro-colorimeter calibrated with glucose standard. The results were expressed as mg glucose 100 g^− 1^ dry weight.

### Total carbohydrates

Total carbohydrates percentages were estimated according to Albalasmeh [40]. A known weight of dried tissue (0.2–0.5 g) was mixed with 10 mL of sulphuric acid (1 N) and kept overnight in an oven at 100 °C. The solution was then filtered and completed to a known volume. Then one ml of sugar solution was mixed with 1 ml of 5% aqueous phenol solution followed by 5.0 ml of concentrated sulphuric acid. Then the tubes were thoroughly shaken for 10 min and kept in a water bath at 23–30 °C for 20 min. The absorbance was recorded at 490 nm.

### Statistical analysis

Analyses of variance (ANOVA) for all data were calculated using SPSS v20.0 (SPSS Inc., Chicago, USA) [41] analyzing software. Statistical significances of the means were compared with Duncan’s test at *p* ≤ 0.05 levels, the standard error (SE) of the means presented in tables, and figures are means ± SE (*n* = 3).

## Results

### Evaluation of morphological parameters

Our obtained results on the changes in growth parameters presented in Table 1 showed that irrigation of wheat plants with 6.25 dSm^-1^ salt water significantly decreased different studied shoot morphological criteria. Shoot length decreased from 53.67 to 40.33 cm, number of leaves per tiller decreased from 4.33 to 3.67, flag leaf decreased from 29.17 to 22.20 and tillers fresh weight decreased from 5.59 g to 4.05 g, compared to the plants grown under normal conditions. Furthermore, salinity stress decreased significantly root length (cm), and fresh weight (g). Root length decreased from 13.67 to 11.67 cm, and root fresh weight decreased from 1.80 g to 1.41 g. On the other hand, foliar application of wheat plants with different concentrations of TRIA (25, 50, 75, and 100 µM) improved all the studied growth parameters, comparing to the untreated control plants. These increases in growth parameters were gradual and significant till 75 µM, then decreased slightly with 100 µM TRIA level. Furthermore, under salinity stress conditions, TRIA with all used levels mitigated the reduced salinity stress impact via improving those morphological parameters, namely shoot length, number of leaves per tiller, flag leaf area, shoot fresh and dry weight, in addition root length, fresh, and dry weight compared with the control plants (Table 1). However, 75 µM TRIA treatments caused the greatest increases of those parameters regarding all treatments and control, either under normal or stressed conditions.

**Table 1:** Influence of TRIA (0, 25, 50, 75 and 100 µM) on growth parameters of wheat plant exposed to salinity stress (0.23 dS m^− 1^ and 6.25 dS m^− 1^). (Results are average of two seasons)

Salinity dsm^− 1^	Shoot length (cm)		Leaves no/tiller		Flag leaves area (cm^2^)		Tiller fresh wt (g)		Root length (cm)		Root fresh wt (g)	
TRIA (µM)	0.23	6.25	0.23	6.25	0.23	6.25	0.23	6.25	0.23	6.25	0.23	6.25
0	53.67 ± 5.03e	40.33 ± 5.03 g	4.33 ± 1.15 cd	3.67 ± 1.15d	29.17 ± 1.88e	22.20 ± 3.19 g	5.59 ± 0.42e	4.05 ± 0.18 g	13.67±1.15e	11.67±1.15f	1.80±0.21ef	1.41±0.11g
25	64.67 ± 3.06c	47.67 ± 1.15f	4.67 ± 1.15bc	4.33 ± 1.15 cd	34.28 ± 1.87c	27.00 ± 2.85f	6.56 ± 0.52c	5.15 ± 0.16f	16.33±1.15bc	13.33±1.15e	1.90±0.13de	1.75±0.20f
50	72.00 ± 2.00b	54.67 ± 3.05e	5.33 ± 1.15ab	4.67 ± 1.15bc	38.86 ± 1.13b	32.04 ± 1.05d	8.09 ± 0.72b	5.80 ± 0.29de	17.33±3.06ab	15.33±2.31cd	2.11±0.12bc	1.92±0.13de
75	75.33 ± 1.15a	59.33 ± 2.31d	5.67 ± 1.15a	4.67 ± 1.15bc	42.66 ± 1.88a	34.26 ± 1.06c	9.19 ± 0.68a	5.92 ± 0.23de	18.33±1.15a	17.00±2.00ab	2.47±0.32a	2.23±0.16b
100	64.00 ± 4.00c	49.67 ± 1.15f	5.33 ± 1.15ab	4.33 ± 1.15 cd	34.34 ± 1.23c	27.28 ± 1.28f	6.14 ± 0.33d	4.92 ± 0.36f	16.67±1.15bc	14.00±2.00de	1.98±0.04cd	1.73±0.22f

± indicate standard deviation of three replicates. Means followed by the same letter were not significantly different at *P* < 0.05 in each parameter

### Evaluation of photosynthetic pigments

**Fig. 3:**
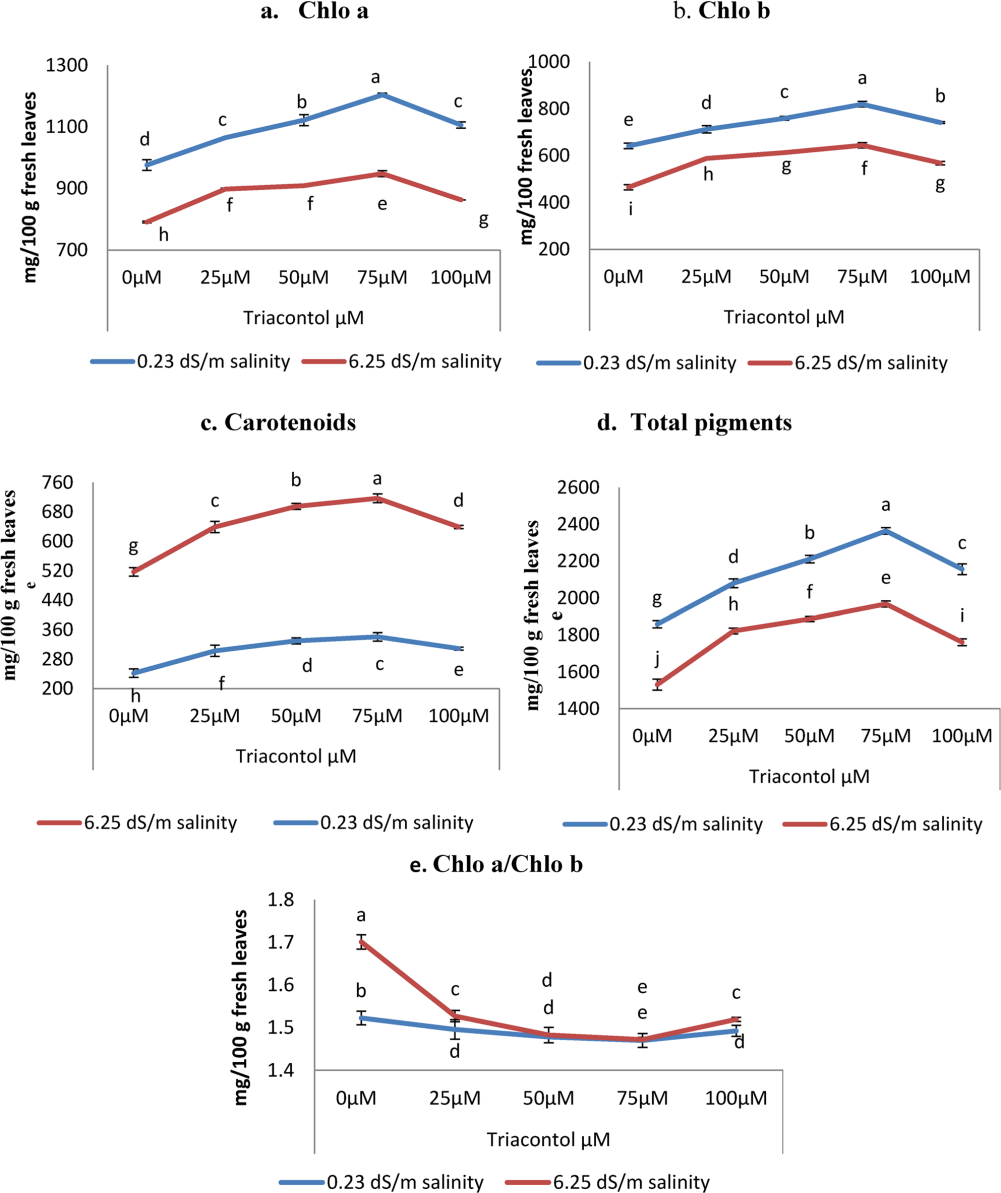
Influence of TRIA (0, 25, 50, 75 and 100 µM) on (**a**) Chl a, (**b**) Chl b, (**c**) carotenoids, (**d**) total pigments (mg/100 g fresh wt) and (**e**) Chl a/Chl b ratio of wheat plant exposed to salinity stress (0.23 dS m^-1^ and 6.25 dS m^-1^). ± indicate standard deviation of three replicates. Means followed by the same letter were not significantly different at P<0.05 in each parameter

A trait-like morphological pattern was also observed. The contents of photosynthetic pigments decreased significantly with 6.25 dSm^− 1^ salt concentrations (Fig. 3) as compared with control plants. The concentration of chlorophyll (Chlo a) decreased by 18.95%, Chlo b declined by 27.46%, and total pigments decreased by 17.64%, while carotenoids content and the ratio of Chlo a/ Chlo b increased significantly by 13.64% and 11.72%, respectively as compared with the unstressed control plant. While, triacontol treatment under normal irrigation water improved Chlo a, Chlo b, carotenoids and consequently total pigment contents in addition to the ratio of chlorophyll a to chlorophyll b as compared with untreated control. Furthermore, Triacontol treatments with different levels displayed positive influence by significant increases in Chlo a, Chlo b, carotenoids, and consequently total pigments, however, it decreased significantly the Chlo a/Chlo b ratio regarding untreated plants (Fig. 3).

### Evaluation of endogenous Indole Acetic Acid (IAA)

**Fig. 4:**
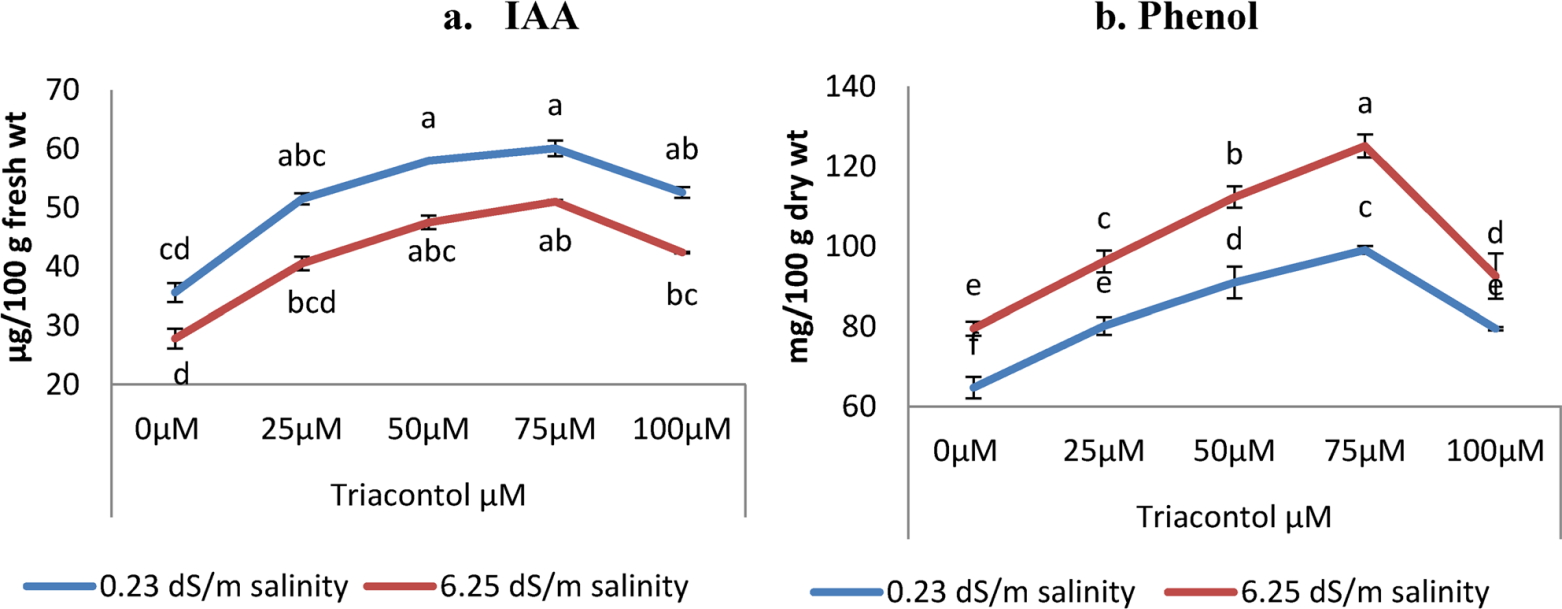
Influence of TRIA (0, 25, 50, 75 and 100 µM) on (**a**) endogenous IAA (µg/100 g fresh wt) and (**b**) phenol content (mg/100 g dry wt) of wheat plant exposed to salinity stress (0.23 dS m^− 1^ and 6.25 dS m^− 1^). ± indicate standard deviation of three replicates. Means followed by the same letter were not significantly different at *P*<0.05 in each parameter

Statistical analysis showed significant differences in endogenous indole acetic acid IAA (Fig. 4a) among different treatments. Salinity (6.25 dS m^− 1^) reduced the IAA content by 21.86% as compared to the control plants grown in normal water. TRIA treatment under normal irrigation and salinity stress displayed a positive effect on the IAA contents with all applied concentrations (25, 50, 75, and 100 µM) as plants exhibited significant increases of wheat plants compared to untreated controls. These increases in IAA contents were gradual and significant till 75 µM, then decreased slightly with 100 µM TRIA level (Fig. 4). However, 75 µM TRIA treatments gave the greatest increments of IAA parameter, regarding all treatments and controls, either under normal or stressed conditions.

### Evaluation of total phenol content

Statistical analysis showed significant differences in total phenol content (Fig. 4b). Salinity (6.25 dS m^− 1^) increased total phenol content by 22.83% as compared to the plants grown in normal water. Triacontol treatment under normal irrigation and salinity stress displayed positive effect on the phenol contents with all applied concentrations (25, 50, 75, and 100 µM) as plants exhibited a significant increase of wheat plants as compared to untreated controls. These increases in phenol contents were gradually and significantly till 75 µM then decreased slightly with 100 µM TRIA level (Fig. 4). However, 75 µM TRIA treatments gave the greatest increment of phenol content regarding all treatments and control either under normal and stressed conditions.

### Evaluation of hydrogen peroxide H_2_O_2_

**Fig. 5:**
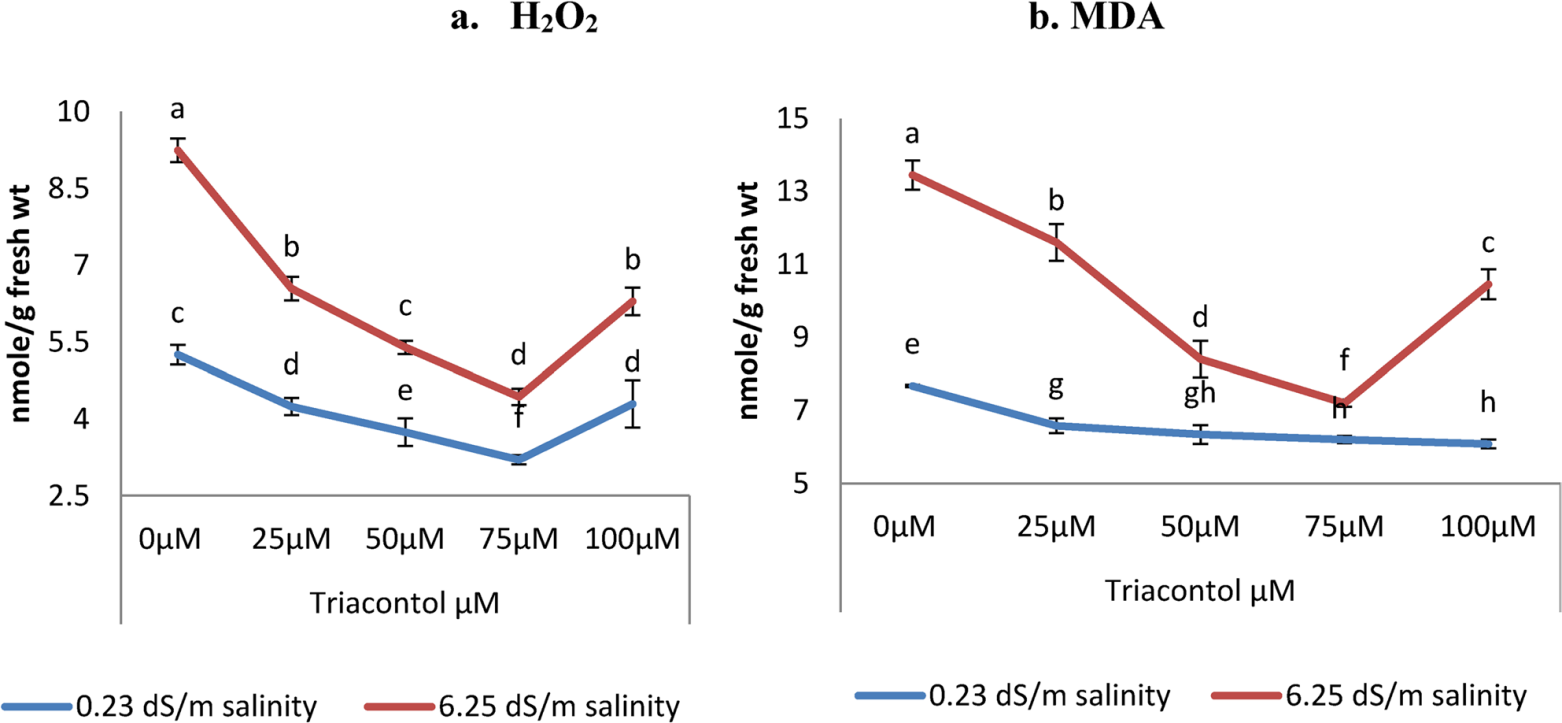
Influence of TRIA (0, 25, 50, 75 and 100 µM) on (**a**) hydrogen peroxide and (**b**) MDA (nmole/g fresh weight) of wheat plant exposed to salinity stress (0.23 dS m^− 1^and 6.25 dS m^− 1^). ± indicate standard deviation of three replicates. Means followed by the same letter were not significantly different at *P* < 0.05 in each parameter

Hydrogen peroxide (H_2_O_2_) significantly increased in wheat leaves under salt stress conditions (Fig. 5). Salinity stress (6.25 dS/m) increased H_2_O_2_ content by 76.0% compared with control plants. Meanwhile, foliar application of TRIA (25, 50, and 100 µM) reduced significantly H_2_O_2_ of salt-stressed and non-stressed wheat plants. Furthermore, H_2_O_2_ content decreased consistently in the salt-stressed plants with an increase in the level of TRIA applied. Treatment of wheat plant with 75 µM TRIA was the most effective concentration over other used concentrations and control treated plants (Fig. 5). The percentages of decreases reached 39.05% and 52.06% under normal and stressed conditions respectively.

### Evaluation of malondialdehyde (MDA)

The obtained data in Fig. 5 show that, irrigation of wheat plant with 6.25 dS/m salt water increased significantly malondialdehyde (MDA) regarding to control plants (0.23 dS/m). Meanwhile, foliar application of TRIA with different concentrations (25, 50 and 75 µM) significantly decreased the MDA content under both salt levels (0.23 and 6.25 dS/m), and 75 µM TRIA was the most impacted on wheat plants (Fig. 4).

### Evaluation of antioxidant enzyme activity

**Fig. 6:**
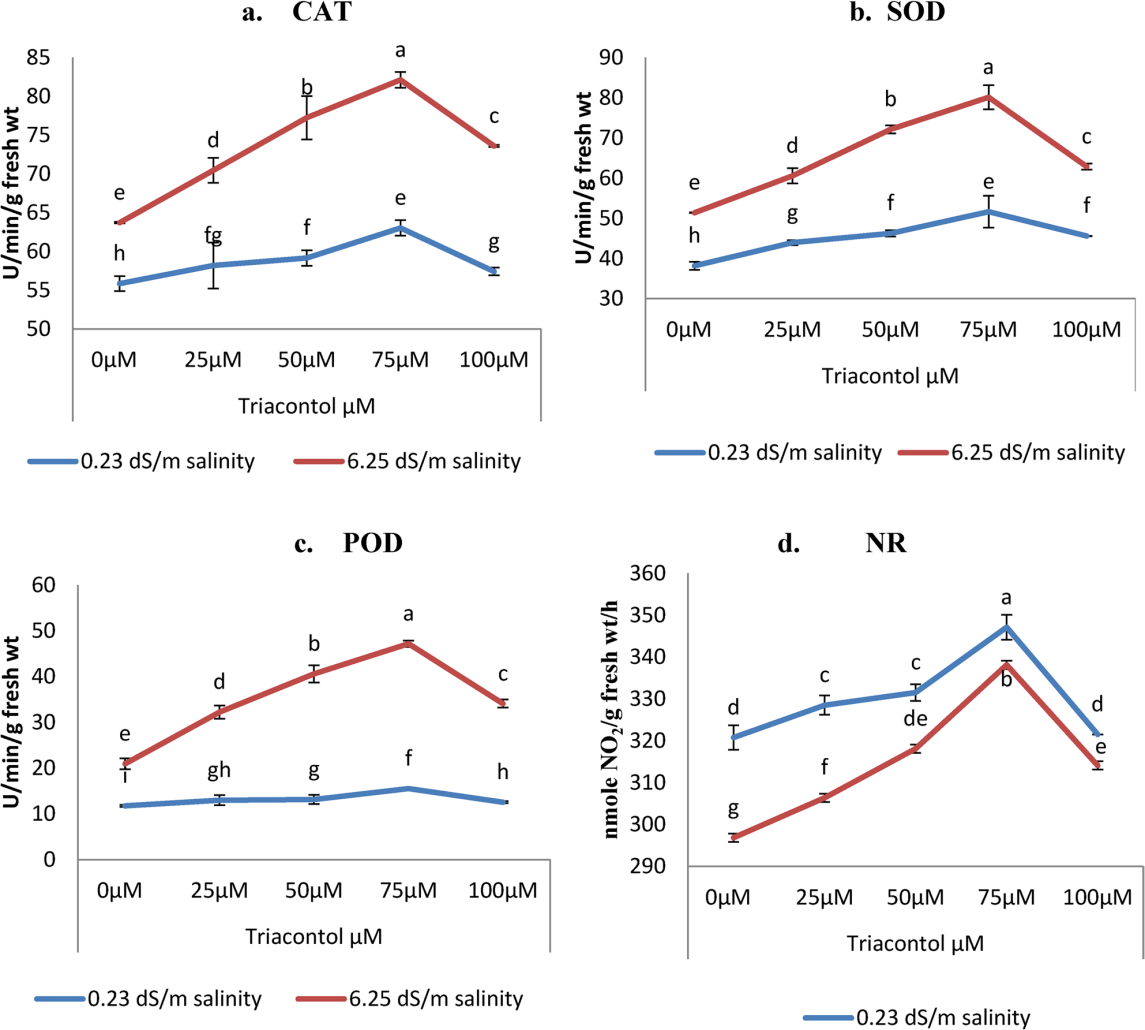
Influence of TRIA (0, 25, 50, 75 and 100 µM) on antioxidant enzymes (**a**) CAT, (**b**) SOD, (**c**) POD (U/min/g fresh wt) and (**d**) NR (nmoleNO_2_/g fresh wt/h) of wheat plant exposed to salinity stress (0.23 dS m^− 1^ and 6.25 dS m^− 1^). ± indicate standard deviation of three replicates. Means followed by the same letter were not significantly different at *P*<0.05 in each parameter

Results of this investigation stated that salinity stress with (6.25 dS/m) significantly increased some enzymatic antioxidant activities namely; catalase (CAT), superoxide dismutase (SOD), & peroxidase (POD) (Fig. 6a, b, c) relative to control unstressed wheat plant (0.23 dS/m). While, stress improved CAT activity (55.84 to 63.70 U/min/g fresh weight), SOD (38.18 to 51.39 U/min/g fresh weight), POD (11.75 to 20.94 to 173.0 U/min/g fresh weight), regarding unstressed plants. On the other hand, all applied concentrations of TRIA (25, 50 and µM) significantly increased activity of CAT, SOD, and POD in fresh leaf tissues of wheat plant grown either under unstressed or salt stressed conditions relative to their corresponding controls. The most pronounced treatments were 75µM of TRIA wheat treated plant either under unstressed or stressed conditions (Fig. 6).

### Evaluation of nitrate reductase enzyme activity

The obtained data of Fig. 6d show that subjecting wheat plant to salinity stress (6.25 dS/m) decreased significantly nitrate reductase (NR) enzyme activity compared with unstressed plant. Since, it decreased from 320.77 to 296.84 nmole NO_2_/g fresh wt/h, relative to control plant. While TRIA treatments with different concentrations caused significant increases in NR activity under both irrigation water (Tap water and salt water) compared with untreated controls (Fig. 6). 75µM of TRIA wheat treated plant either under unstressed or stressed conditions (Fig. 6).

### Evaluation of primary metabolites

**Fig. 7:**
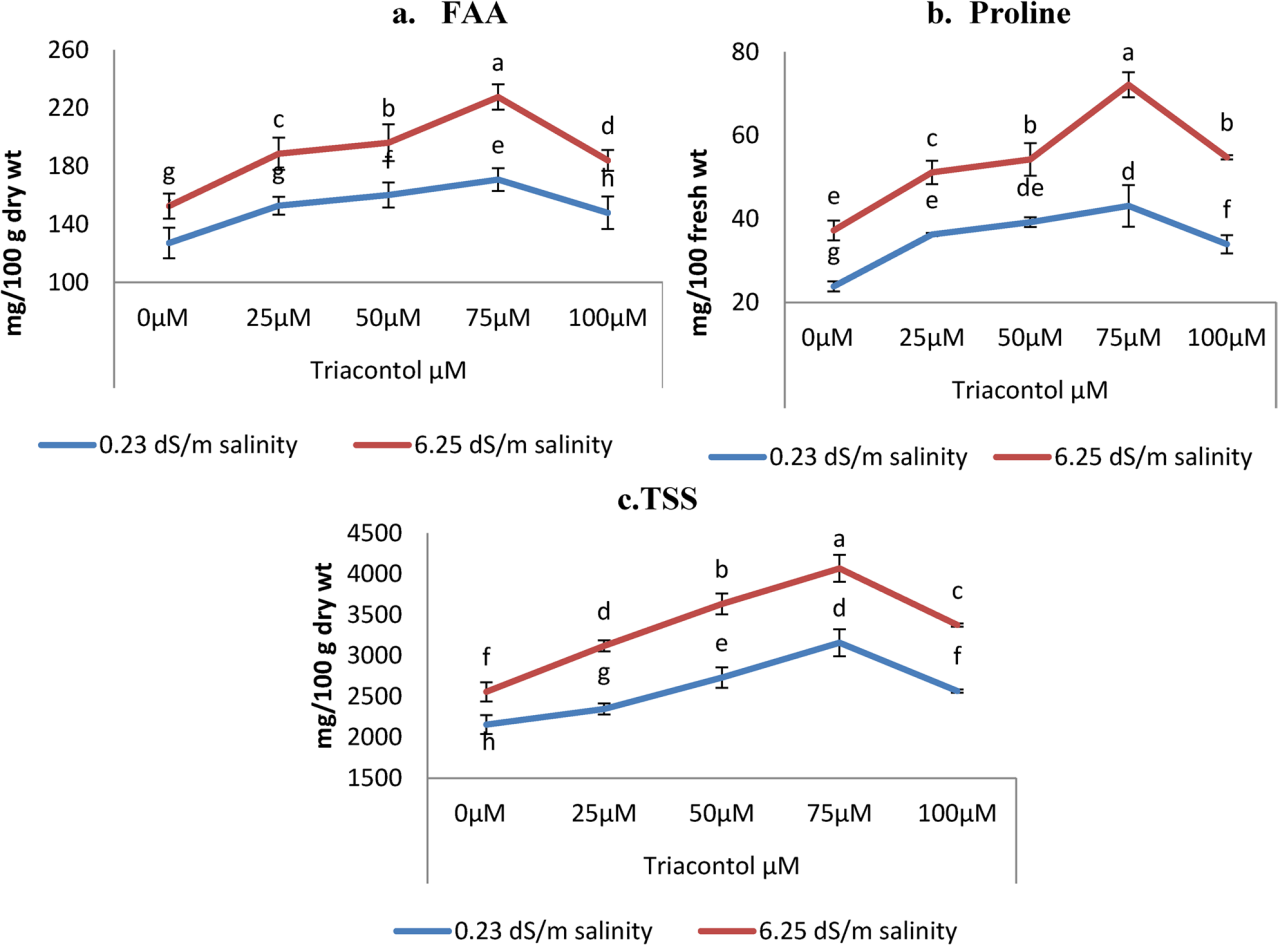
Influence of TRIA (0, 25, 50, 75 and 100 µM) on primary metabolites (**a**) FAA, (**b**) proline and (**c**) TSS mg/100 g dry wt of wheat plant exposed to salinity stress (0.23 dS m^− 1^ and 6.25 dS m^− 1^). ± indicate standard deviation of three replicates. Means followed by the same letter were not significantly different at *P*<0.05 in each parameter

Primary metabolites (free amino acid, proline and total soluble sugars TSS) showed significant increases in their values with increasing salinity levels 6.25 dSm^− 1^ (Fig. 7a, b, c). Statistical analysis showed significant differences in free amino acids, proline and total soluble sugars. Salinity increased the free amino acids by 11.27%, proline by 56.25% and total soluble sugars by 18.65% respectively, as compared to control plants (0.23 dSm^− 1^). Triacontol foliar application under salinity (6.25 dSm^− 1^) displayed positive effect on primary metabolites at all applied concentrations (25, 50, 75 and 100 µM ) as plants exhibited a significant incline in free amino acids, proline and total soluble sugars as compared to control plants normal and salt stress conditions. The results in Fig. 5 showed that 75 µM was the most effective concentration in increasing the above mentioned parameters not only under normal irrigation water but also under salt stress as it caused 34.33% and 49.15% increase of free amino acids, 80.87% and 93.64% of proline and 46.54% and 59.09% under normal and salt stress irrigation water respectively. The obtained data clearly show that the percentages of increases were higher under stress than under normal water (Fig. 7).

### Evaluation of yield and its components

Salinity stress (6.25 dS/m) significantly reduced the yield and its components of wheat plant (such as plant height cm, spike length cm, and weight g, shoot and grains weight g, and 1000 grains weight g) as compared with unstressed control plants (Table 2). Furthermore, total carbohydrates of the yielded grains reduced significantly in plants irrigated with 6.25 dS/m salt water in relation to unstressed plants (0.23 dS/m). Meanwhile, Triacontanol (25, 50 ad 75 µM) applications increased the studied yield parameters under normal and salt stress condition compared with untreated corresponding controls (Table 2).

**Table 2:** Influence of TRIA (0, 25, 50, 75 and 100 µM) on yield and its components and total carbohydrates of the yielded grains of wheat plant exposed to salinity stress (0.23 dS m^− 1^and 6.25 dS m^− 1^). (Data are means of two seasons)

Salinity dsm^− 1^	Shoot length (cm)		Spike length (cm)		Spike wt (g)		Shoot wt (g)		Grains wt (g)		1000 grains wt (g)		1000 grains wt (g)	
TRIA (µM)	0.23	6.25	0.23	6.25	0.23	6.25	0.23	6.25	0.23	6.25	0.23	6.25	0.23	6.25
0	63.3 ± 1.15 g	52.0 ± 3.64 h	9.33 ± 0.65d	8.00 ± 0.86e	2.63 ± 0.49b-d	1.97 ± 0.25d	4.81 ± 0.16e	3.87 ± 0.19 g	1.80±0.21f	1.16±0.09g	35.38±1.29e	29.85±2.16g	45.84±0.37e	43.45±0.40g
25	70.0 ± 3.46e	63.3 ± 3.06 g	11.67 ± 0.75c	9.67 ± 0.95d	3.51 ± 0.19a-c	2.19 ± 0.17d	6.10 ± 0.15b	4.52 ± 0.16ef	2.23±0.12c	1.75±0.12f	38.50±0.67d	32.30±1.36f	46.90±0.10c	44.90±0.44f
50	79.3 ± 3.06b	66.7 ± 4.16f	13.33 ± 0.55b	11.33 ± 1.15c	3.88 ± 0.25ab	2.46 ± 0.18 cd	6.53 ± 0.21b	5.11 ± 0.31d	2.43±0.09b	1.93±0.08e	42.97±2.74b	34.88±0.74	48.81±0.31b	45.90±0.44e
75	86.3 ± 1.15a	72.0 ± 2.00d	15.00 ± 0.80a	13.33 ± 1.15b	4.11 ± 0.09a	2.95 ± 1.34bc	7.44 ± 0.32a	5.30 ± 0.19bc	2.61±0.17a	2.10±0.15d	44.25±1.09a	38.28±0.70d	50.39±0.51a	46.44±0.37d
100	76.7 ± 2.31c	62.3 ± 2.30 g	14.00 ± 1.00ab	11.67 ± 1.55c	3.20 ± 0.15a-d	2.25 ± 0.17 cd	5.62 ± 0.14c	4.44 ± 0.12ef	2.29±0.24c	1.25±0.02g	39.36±0.71c	33.32±0.83f	46.99±0.07c	45.18±0.12f

± indicate standard deviation of three replicates. Means followed by the same letter in each parameter    were not significantly different at *P*<0.05 in each parameter

## Discussion

Crop losses caused by abiotic stress factors have garnered significant attention in research agendas. Numerous studies indicate that such losses can reach up to 40%, depending on the stress type. Of all abiotic stressors, salinity is one of the most severe, drastically reducing crop productivity through osmotic stress, ionic toxicity, and nutritional imbalances [42]. Such perturbations are clearly translated into the reduced water uptake, disrupted photosynthesis and stunted growth. In order to safeguard the crop productivity and quality of the crops and to shield the environment, identifying the strategies regarding alleviating the negative effects is thus a crucial task. In this context, we here explored the comparative effects of TRIA on a serious number of morphological, physiological and biochemical attributes of the wheat plants suffering from salinity stress [43]. We hypothesized that salt stress-mediated losses and impairment would be buffered by the treatments by enhancing the antioxidant system, modulating the stress indices and shifting the metabolism of the secondary metabolite production. Accordingly, as expected, salt stress critically reduced the key growth parameters such shoot length (cm), leaves number per tiller, flag leaves area (cm^2^),tiller fresh ad dry weight (g), root length (cm), root fresh ad dry weight (g) as evidenced in former reports [44, 45]. These decreases are likely due to water and ionic imbalances in cells due to toxic ions. Also, salinity stress induces metabolic perturbation by generating ROS, leading to oxidative stress in plants that causes lipid peroxidation and DNA damage [46].

Meanwhile, TRIA boosted the growth of salt-stressed wheat plants and lessened the impact of salinity. Aziz and Shahbaz [47] stated that TRIA boosted shoot and root growth in sunflowers under salt stress. Application of TRIA improved the studied growth parameters via improving osmotic potential in soybean plants to restore metabolic processes in salt stress [48]. The increase in plant growth by TRIA might be due to the increase in the size and number of the chloroplasts, which in turn increases the chlorophyll and carotenoid contents, thus enhancing the photosynthetic CO_2_ assimilation in plants [24]. TRIA-mediated increases in growth could be due to its role in modulating the activities of different enzymes and enhancing photosynthetic rate [49]. TRIA elicits a second messenger 9-β-L (+) adenosine, which is structurally similar to cytokinins, plant hormones, reported to be actively involved in the promotion of growth and other attributes in plants [50].

Photosynthesis is vital physiological process operating in green plants and generally depends on photosynthetic pigments, gas exchange parameters, photosystems, components of electron transport systems and enzymes involved in carbon metabolism Wheat plats grown under salt stress showed significant decreases in photosynthetic pigments components, meanwhile, increased significantly carotenoids contents (Fig. 3). Earlier, Sadak [51] were also reported decreases in Chlo a, Chlo b and total pigments while, increased the synthesis of carotenoids content, which are considered non-enzymatic antioxidants, and enhanced the reduced oxidative damage of white termis. High levels of Na ^+^ reduce the functioning of CO2 fixing enzymes, and the tolerance potential of these enzymes to high Na^+^ ions differs in various plant species [52]. Furthermore, the proton motive force gets altered, which impacts the photosynthetic apparatus and functioning of chloroplast under high Na^+^ concentrations [53]. Photosynthetic attributes such as chlorophyll, photosystems, net photosynthesis rate, ribulose bisphosphate carboxylase/oxygenase (Rubisco activity) etc., are adversely affected by the salinity stress [54]. Therefore, alleviation of salinity induced photosynthetic damage is one of the crucial steps in plants. Also, salinity stress improved proline biosynthesis resulted in less efficient use of glutamate as a precursor in biosynthesizing chlorophyll molecules [55]. Because chlorophyll b degrades more quickly than chlorophyll a, leaves exposed to moderate and salinity stress had lower chlorophyll concentrations with greater chlorophyll a/b ratios. This could be explained by the fact that chlorophyll b degradation is the first step [56]. Carotenoids have been found to play a significant function in regulating photoprotection against photooxidation [57] and serving as antioxidant that scavenge reactive oxygen species (ROS) to avoid the creation of ^1^O_2_ [56].

Applying TRIA foliar spraying greatly reduced the negative effects of salinity in *Triticum aestivum* on photosynthetic pigments constituents (Fig. 2). This benefit was achieved by enhancing the performance of the constituents of the photosynthetic apparatus, including chlorophyll levels, membrane leakage, photosynthetic rate, stomatal conductance, and electron transport rate [57]. Similar results with higher chlorophyll a, chlorophyll b, and carotenoids levels with TRIA were seen in *Brassica napus* under saline circumstances [58]. The larger and more numerous chloroplasts, which raise the concentrations of carotenoid and chlorophyll to improve photosynthetic CO_2_ assimilation in plants, may be the cause of the increased plant growth caused by TRIA [23, 59]. Overall, it was stated that the use of TRIA prevent the deprivation of photosynthetic apparatus via improving the content of photosynthetic pigments and increasing the expression of PSI, PSII, and other photosynthesis-related proteins. Using TRIA significantly reduced the negative impacts of salinity by promoting Rubisco activity that might have resulted in improving the Calvin cycle functioning [60].

The earlier findings demonstrated the lower endogenous auxin levels (measured in relation to an unstressed control group). Sadak and Dawood [61] and Sadak [51] previously verified these findings on wheat and white lupine plants. The decrease in IAA content resulted from the disruption of the enzyme activity involved in the formation of IAA in plant cells [62]. Accordingly, the disruption in the hormonal balance could be the reason for reduced plant growth brought on by stress conditions. In the meantime, the data shown in Fig. 3 indicated that IAA was elevated in response to triacontol. Similar outcomes were observed in Pang’s study on strawberries [63]; TRIA may have a stimulating effect on IAA content by reducing its degradation by lowering IAA-oxidase, increasing the amount of free IAA, and lowering the conversion of active free auxin to inactive.

Phenols are known for their excellent antioxidant properties, which have an important impact on scavenging reactive oxygen species, thus improving plant tolerance against salinity [64]. Therefore, these compounds experience various adaptive mechanisms in response to stressful situations [65]. Plants experiencing osmotic shock due to salinity stress produced more phenols in the plant. Ghasemzadeh and Ghsemzadeh [66]. Additionally, phenols reduce the release of reactive oxygen species (ROS) thus decreasing peroxidation events by inhibiting lipid peroxidation and increasing membrane integrity and fluidity [67]. Furthermore, TRIA foliar treatment to wheat with different concentrations increased its total phenols content. This work supports the findings of Ertani [67] and Shaguftap [68], who demonstrated that phenol content in salt-stressed maize and wheat plants, was reduced by a biostimulant based on TRIA.

Salinity harms plants by causing ionic toxicity, osmotic stress, inadequate nutrition, and genotoxicity, which results in oxidative stress and reactive oxygen species (ROS) overproduction [24]. Increased levels of ROS accumulation are harmful to the integrity of cells because they can lead to oxidative stress-induced damages like lipid peroxidation, which is caused by the synthesis of malondialdehyde (MDA) and thiobarbituric acid-reactive substances, as well as denaturation of DNA, proteins, lipids, and other cell components [69]. Earlier studies of Sadak [70] confirmed the increasing levels of hydrogen peroxide and lipid peroxidation of wheat plants under salt stress. On the hand, the application of TRIA repressed lipid peroxidation in spinach (*Spinacia oleracea* L.) [71] and in leaves of *Arachis hypogaea* L., which in turn improved the integrity of membranes [72].

To keep plants’ metabolisms functioning normally in the face of salt stress, plant generate a variety of divergent signals related to ROS [73]. Therefore, cellular ROS balance must be maintained by regulating their production and scavenging, which is maintained by antioxidant defense system. In our results, antioxidant enzymes (CAT, SOD, POD and NR) increased due to salinity stress, which is in harmony with a previous study obtained El-Lethy [74] and Khattab [75], they stated that salinity stress significantly increased CAT, SOD and POD enzyme activities and lowered H_2_O_2_ and MDA levels of wheat and flax plants, respectively. Under stress conditions, TRIA acts an antioxidant substance, as it improve antioxidant enzymes activities on one hand and reduces lipid peroxidation on the other. For example, exogenous treatments of 10 and 20 µM TRIA decreased the oxidative stress-induced membrane damage in wheat cultivars via markedly increased peroxidase (POD) activity and a consequent reduction in the H_2_O_2_ and MDA levels [76]. Suman [77] stated that the negative effects of salinity stress were lessened by TRIA, which increased the activity of the enzymes POD, SOD, and glutathione reductase (GR) rice plant. Further, foliar sprays of 50 and 100 µM TRIA ameliorated the harmful effects of salinity by up-regulating the activities of POD, superoxide dismutase (SOD) and glutathione reductase (GR) enzymes in sunflower [47]. However, Karam & Karamat [16] found that via modifying the activity of the enzymes MDA, H_2_O_2_, CAT, ascorbate peroxidase (APX), SOD, and POD, foliar sprays of 10 and 20 µM TRIA were successful in lessening the harmful effects of salinity stress on *Coriandrum sativum* L. Furthermore, four sprays of 2 and 5 µM TRIA each enhanced salinity tolerance by notably lowering the relative membrane permeability, H2O2, and MDA levels in maize seedlings, according to Perveen [49]. Furthermore, Khanam and Mohammad [50] recently investigated the impact of TRIA on *Mentha piperita* L. antioxidant systems at varying salinity levels. By significantly raising CAT, POD, and SOD activity, foliar sprays of 1 µM TRIA mitigated the impacts of salt. Increased antioxidant enzyme activity has been credited with TRIA’s ability to reduce the salt stress effect. This detoxification of ROS led to a balance between ROS generation and scavenging, which in turn reduced the harmful effects of salinity on plants and, ultimately, affected plant growth [16]. Thus, it can be inferred from the discussion above that the application of TRIA can reverse the damage caused by salt in plants that have been tested by modifying the antioxidant defence system, which offers hints to identify the underlying mechanisms that give other plants, in addition to the tested plants, the ability to withstand salt stress.

Osmolyte production is a typical phenomenon in stress mitigation to lessen physiological harm. This study stated that wheat plants accumulated more free amino acids, proline, and total soluble sugar levels (Fig. 6), which was a self-regulating mechanism of plants in response to salinity stress. These obtained results, are confirmed on various plant species under stress [78, 79]. According to Oraki [80] these increases in TSS and proline may aid plant cells in controlling their osmotic potential, which would enhance their ability to absorb and translocate water in salty environments. Proline also functions as a scavenger of elevated levels of free radicals and protects many cell components and enzymes from oxidative damage. Proline uses two methods to lessen the damage caused by ROS: chemical interaction with OH radicals and singlet oxygen (^1^O_2_) quench [81]. Furthermore, the greater proline content is caused by an inhibition in proline oxidase activity. Nonetheless, in terms of quick recovery and stabilisation, it is regarded as a C and N donor. In both saline and normal environments, exogenous application of TRIA significantly increased the levels of osmolytes in wheat plants, including proline, free amino acids, and total soluble sugars [82]. In both our study (Fig. 7) and a prior work by Alharbi [83] and El-Beltagi [84] exogenous applications of TRIA resulted in a significant increase in proline under abiotic stress (salinity and drought). Additionally. according to Ries [85], TRIA caused the treated plants’ levels of free amino acids and soluble sugars to rise at the same time. TRIA treatment also markedly raised the sugar content and TSS of the leaves of *Bougain villaea* plants in a different research [51].

**Fig. 8:**
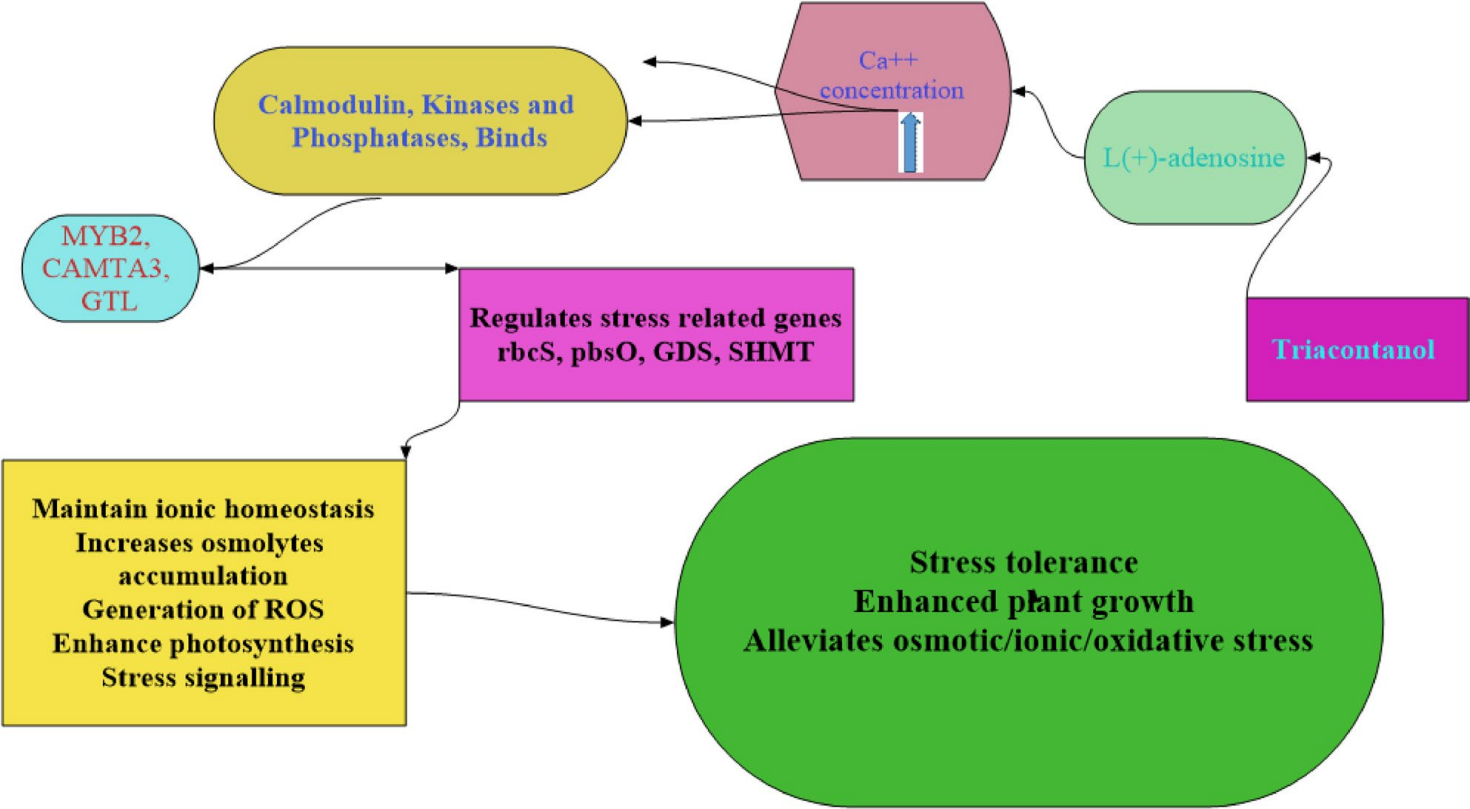
Mode of action of triacontanol under stress conditions [20]

Our findings showed that, salinity stress significantly reduced yield attributes and carbohydrate contents of wheat plant (Table 2). Those reductions might be due to growth (Table 1) and photosynthetic pigment reductions (Fig. 2). Additionally, chlorophylls reductions caused photosynthesis activity decreases, low carbohydrate biosynthesis, and reduced translocation from leaves to seeds [86]. Salinity stress negatively impacted enzymatic activities, thus decreasing growth and yield attributes [79]. Meanwhile, TRIA treatment enhanced yield attributes and carbohydrate contents of wheat plant (Table 2). In accordance with our obtained results, Shahbaz [87] found that pre-sowing TRIA enhanced the yield quantity quality of *Brassica napus* L. under salt circumstances. Perveen [82] also found that under salt stress, TRIA applied to the leaves significantly enhanced the weight of the seeds, the number of grains, and the yield per plant of wheat. Furthermore, under salinity conditions, Khanam and Mohammad [50] observed that at 1 µM TRIA spray enhanced the yield of *Mentha piperita* L. According to Khan [88], treating *Solanum lycopersicum* L. leaves with a low concentration of TRIA increased yields. Earlier efforts have been made to understand the mechanism of TRIA activation [10]. TRIA is derived from Trim, specifically 9-β-l(+)-adenosine, also known as 9 H-purine-6-amine, 9-βL-ribofuranosyl. These findings were instrumental in discovering TRIA. It was found that TRIA produces a secondary messenger (TRIM) in rice plants [85]. This messenger induces the same plant responses as TRIA but at nanomolar levels [89].The initial step in TRIA’s action in plants was identifying TRIA as a TRIM by producing L(+)-adenosine. The spray treatment of TRIA also promoted specific physiological characteristics, including malate dehydrogenase activity and plant growth [89]. Increased L(+)-adenosine concentration leads to an elevation in Ca^++^ concentration within the tonoplast. This rise in Ca^++^ levels hinders the action of specific transcription factors like calmodulin protein, MYB2, CAMTA3, GTL, and others. At the same time, it enhances the function of kinases and phosphatases, thereby promoting the activation of photosynthetic genes and other related genes as illustrated in Fig. 8.

## Conclusions

Recently, there has been an expansion in the use of TRIA as an antioxidant combination for improving salinity plant tolerance; Triacontol treatment has the ability to minimize the reductions of plant growth and productivity of wheat plants via improving various physiological aspects. More molecular studies are needed for understanding the mechanism of Triacontol in enhancing plant tolerance to abiotic stresses. Finally, supplementary applications of Triacontol with 75 µM on wheat plants are recommended to minimize the damage of salinity stress, which could be useful for commercial production and the private sector.

## Data Availability

The authors declare that all data generated or analyzed during this study are included in this published article.
